# Complex and simple translational readthrough signals in pea enation mosaic virus 1 and potato leafroll virus, respectively

**DOI:** 10.1371/journal.ppat.1010888

**Published:** 2022-09-29

**Authors:** Tamari Chkuaseli, K. Andrew White

**Affiliations:** Department of Biology, York University, Toronto, Ontario, Canada; Iowa State University, UNITED STATES

## Abstract

Different essential viral proteins are translated via programmed stop codon readthrough. Pea enation mosaic virus 1 (PEMV1) and potato leafroll virus (PLRV) are related positive-sense RNA plant viruses in the family Solemoviridae, and are type members of the Enamovirus and Polerovirus genera, respectively. Both use translational readthrough to express a C-terminally extended minor capsid protein (CP), termed CP-readthrough domain (CP-RTD), from a viral subgenomic mRNA that is transcribed during infections. Limited incorporation of CP-RTD subunits into virus particles is essential for aphid transmission, however the functional readthrough structures that mediate CP-RTD translation have not yet been defined. Through RNA solution structure probing, RNA secondary structure modeling, site-directed mutagenesis, and functional in vitro and in vivo analyses, we have investigated in detail the readthrough elements and complex structure involved in expression of CP-RTD in PEMV1, and assessed and deduced a comparatively simpler readthrough structure for PLRV. Collectively, this study has (i) generated the first higher-order RNA structural models for readthrough elements in an enamovirus and a polerovirus, (ii) revealed a stark contrast in the complexity of readthrough structures in these two related viruses, (iii) provided compelling experimental evidence for the strict requirement for long-distance RNA-RNA interactions in generating the active readthrough signals, (iv) uncovered what could be considered the most complex readthrough structure reported to date, that for PEMV1, and (v) proposed plausible assembly pathways for the formation of the elaborate PEMV1 and simple PLRV readthrough structures. These findings notably advance our understanding of this essential mode of gene expression in these agriculturally important plant viruses.

## Author summary

RNA viruses use a variety of strategies to express their encoded proteins, and one mechanism used is translational readthrough of stop codons. Some viruses in the family Solemoviridae use this expression strategy to make a C-terminally extended minor capsid protein that, when incorporated into virus particles, mediates plant-to-plant transmission by aphids. In this study we investigated the RNA sequences and structures that facilitate readthrough production of the minor capsid proteins of pea enation mosaic virus 1 (PEMV1; genus Enamovirus) and potato leafroll virus (PLRV; genus Polerovirus). A combination of structural and functional analyses allowed for the identification of RNA elements that contribute to the readthrough process. In both cases, the RNA structures that facilitate readthrough were formed via long-distance RNA-RNA interactions, with the active readthrough structure for PEMV1 being significantly more complex than that for PLRV. These results provide the first structural models for readthrough signals in these virus genera, and provide new insights into how these critical RNA structures assemble. This information significantly advances our understanding of this important gene expression strategy that is employed by these agriculturally important viruses.

## Introduction

Programmed stop codon readthrough is an alternative protein expression strategy utilized by different viruses. The readthrough process involves the decoding of a stop codon as a sense codon by near-cognate tRNAs, which then allows ribosomes to continue translating in the original reading frame. The resulting C-terminally extended readthrough product is functionally distinct from its pre-readthrough protein, which expands the coding capacity of viral mRNAs. Positive sense RNA viruses represent the largest proportion of the viruses that employ readthrough [1–3]. Some of these viruses, like alphaviruses, alphacarmoviruses, tombusviruses, betanecroviruses, tobamoviruses, etc., [4–9] express their RNA-dependent RNA polymerase (RdRp) via readthrough. Other viruses, such as members of Benyvirus, Furovirus, Pomovirus, Luteovirus, Polerovirus and Enamovirus genera [1,10–14] translate a C-terminally extended minor capsid protein (CP) by means of readthrough.

Readthrough efficiency is promoted and fine-tuned by downstream regulatory RNA sequences and structures [1–3] that are either positioned 3′-proximally to readthrough sites [9] or involve both proximal and distal RNA elements that are united by RNA-RNA interactions [5–8]. Some distal readthrough elements (DRTEs) are separated from their complementary proximal readthrough elements (PRTEs) by shorter distances (~50 to 150 nt), as observed for RdRp production in alphaviruses and predicted for furoviruses, tobraviruses, pecluviruses, and pomoviruses [5]. However, other DRTEs involved in RdRp translation are positioned several thousand nucleotides downstream from their cognate PRTEs, as in viruses in the Tombusvirus, Betanecrovirus, and Alphacarmovirus genera [6–8].

Members of Luteovirus (Tombusviridae), Polerovirus (Solemoviridae) and Enamovirus (Solemoviridae) genera, all of which are related by similarities in their CPs and corresponding readthrough products, have been proposed to regulate CP readthrough from their subgenomic (sg) mRNAs via long-distance RNA-RNA interactions (spanning ~700 nt); based on observed complementarity between corresponding PRTEs and DRTEs [12,13]. Viruses belonging to these three genera are economically important pathogens causing major crop losses of pea, bean, potato and other food crops around the world [15–18]. These viruses require aphid vectors for plant-to-plant transmission and readthrough of the CP stop codon generates a C-terminally extended minor CP, referred to as CP-readthrough domain (CP-RTD), that plays a key role in aphid transmission [14,19–24]. CP-RTD also facilitates other viral events, such as systemic movement in infected plants, persistence of virions in aphid vectors, tissue tropism, and phloem loading [20,21,23,25–31].

To date, only limited information is available about the regulation of readthrough-mediated translation of CP-RTD from sg mRNAs in Luteovirus, Polerovirus and Enamovirus genera [12,13]. The luteovirus barley yellow dwarf virus (BYDV) was the first virus shown to require RNA sequences both proximal (i.e. PRTE) and distal (i.e. DRTE) from its CP stop codon for efficient readthrough [12] and, subsequently, the polerovirus potato leaf roll virus (PLRV) was shown to have similar requirements [13]. Despite the existence of complementarity between the PRTEs and DRTEs in these and other related viruses, no experimental evidence confirming the functional importance of such long-distance interactions has been reported, nor have there been any studies investigating the structural nature of the functional readthrough-promoting RNA signals.

**Fig 1:**
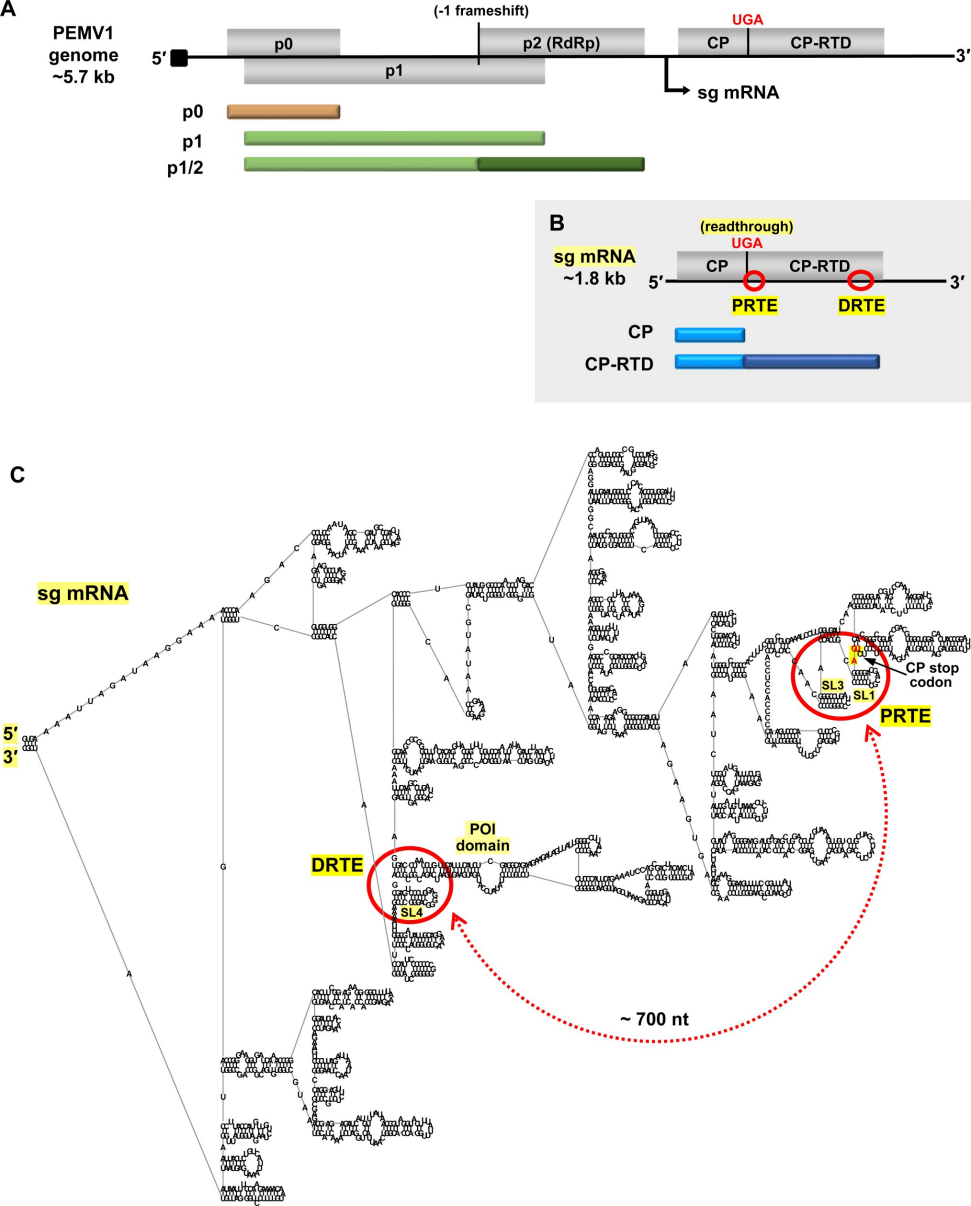
Organization of PEMV1 genome and subgenomic mRNA. **(A)** Linear representation of PEMV1 genome showing encoded ORFs (grey boxes) for p0, p1, p2, coat protein (CP) and coat protein-readthrough domain (CP-RTD). Proteins translated from the genome are shown beneath it as tan and green bars. P1/2 RdRp protein is expressed via programmed -1 frameshifting within the p1 ORF. Black arrow beneath the genome indicates the transcription initiation site for the subgenomic (sg) mRNA. The black square at the 5′-end of the genome represents the VPg. **(B)** PEMV1 sg mRNA encoding CP and CP-RTD. Corresponding translation products are indicated below as blue bars. CP-RTD is expressed via programmed readthrough of the CP UGA stop codon. Relative positions of the proposed readthrough-regulating proximal readthrough element (PRTE) and distal readthrough element (DRTE) are shown as red circles. **(C)** RNA secondary structure model of full-length PEMV1 sg mRNA, as predicted by *RNAStructure* using default settings [34] and rendered using *RNA2Drawer* [57]. Labelled are the 5′ and 3′ ends, PRTE, DRTE, CP stop codon, SL1, SL3, SL4 and the pink-orange intervening (POI) domain. The red circles on the folded structure correspond to the regions circled on the linear sg mRNA in panel B. The proposed long-distance RNA-mediated interaction between PRTE and DRTE is indicated by a red double-headed arrow, and spans approximately 700 nt.

Pea enation mosaic virus (PEMV1) is an enamovirus with a 5.7 kb-long plus-strand RNA genome that contains a 5′ viral protein genome-linked (VPg) and no 3′ poly(A) tail (**Fig 1A**) [32]. The enamovirus protein coding scheme is very similar to that of poleroviruses, like PLRV, except that poleroviruses encode a few additional smaller proteins [18]. The PEMV1 genome codes for three 5′-proximally encoded non-structural proteins, p0, p1, and p1/2 (RdRp, expressed via frameshifting), all of which are translated from the genome (**Fig 1A**) [32]. Structural proteins, CP and its readthrough product CP-RTD, are expressed from a 1.8 kb-long sg mRNA that is transcribed during infections (**Fig 1B**). Similar to poleroviruses and luteoviruses, the sg mRNAs of enamoviruses were predicted to contain complementary readthrough-promoting PRTEs and DRTEs [13]. However, although the interactions proposed for PEMV1 were in the right general areas of the RTD (**Fig 1B and 1C, red circles**), the base-pairing partner sequences that were suggested were not correct [13], as revealed by results presented herein.

In this study we investigated, in detail, the CP readthrough signal in PEMV1’s sg mRNA and determined that it adopts an elaborate RNA structure, which contrasts the simple readthrough structure that was deduced for PLRV. We also confirmed the functional requirement for long-distance RNA-RNA interactions between the PRTEs and DRTEs in both PEMV1 and PLRV. Lastly, we propose putative folding pathways for formation of the complex PEMV1 and simple PLRV readthrough structures.

## Results

### Secondary structure analysis of the PRTE and DRTE in PEMV1 sg mRNA

**Fig 2:**
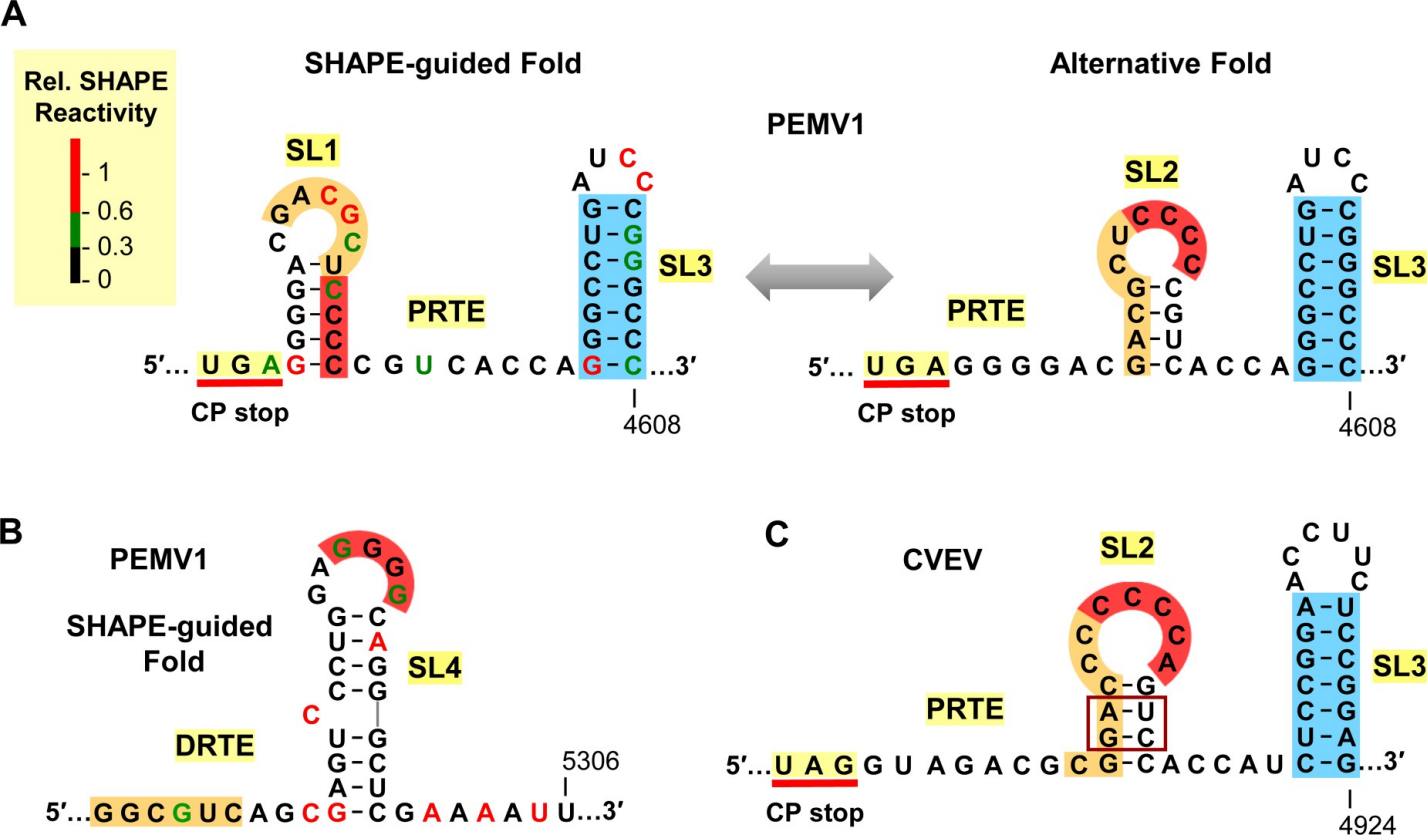
SHAPE-guided RNA secondary structures of PRTE and DRTE. **(A)** SHAPE-guided and alternative folds for the PRTE in PEMV1. Relative SHAPE reactivities of individual nucleotides are colour-coded (see key) in the SHAPE-guided fold (left). The CP stop codon, SL1, and SL3 are labeled. An alternative fold in which SL2 forms is shown on the right, with a double-headed arrow depicting the proposed interconversion between the two conformations. The stem of SL3 is highlighted in blue, while orange and red highlights denote sequences that have complementary partner segments in the DRTE, which is shown in panel B. **(B)** SHAPE-guided fold of DRTE in PEMV1. Nucleotide reactivities are colour-coded and segments complementary to red and orange RNA segments in the PRTE are indicated. **(C)** SL2 and SL3 PRTE equivalents predicted in citrus vein enation virus (CVEV, NC_021564), with corresponding orange, red, and blue segments highlighted.

Prior to investigating the functional involvement of the PRTE and DRTE in regulating PEMV1 CP stop codon readthrough (**Fig 1B and 1C**), the local RNA secondary structures in these regions were analyzed via selective 2′-hydroxyl acylation analyzed by primer extension (SHAPE) [33]. SHAPE was conducted on in vitro synthesized transcripts of the full-length wild type (wt) PEMV1 sg mRNA and the reactivity data gathered (SHAPE reactivity correlates with flexibility of the corresponding nucleotide) were integrated into the *RNAStructure* folding program to predict the most probable secondary structure [34]. The results for the PRTE region revealed the presence of two small RNA stem-loop (SL) structures, termed SL1 and SL3, located downstream of the CP stop codon (**Fig 2A, left**). Interestingly, an alternative fold was also possible for the sequence between the stop codon and SL3, in which SL1 is replaced by a mutually-exclusive SL2 (**Fig 2A, right**). Notably, SL2 contains four cytidylate residues (red) in its terminal loop (**Fig 2A, right**), which are complementary to four guanylate residues (red) in the terminal loop of the SHAPE-predicted SL4 in the DRTE (**Fig 2B**). Consequently, the complementary terminal loops of SL2 and SL4 could potentially engage in a kissing-loop base-pairing interaction and nucleate contact between the PRTE and DRTE. Subsequent to this initial interaction, additional regions of complementarity, such as the identified orange-highlighted segments, would then be able to pair (**Fig 2A and 2B**).

The sequence in the PRTE between the CP stop codon and SL3 is highly conserved in enamoviruses. However, one genus member, citrus vein enation virus (CVEV), was found to differ significantly in this region [35]. In CVEV, a SL2 equivalent with a tandem base pair covariation (boxed) in the center of its stem was predicted, however a corresponding SL1 could not be identified (**Fig 2C**). Additionally, CVEV has a comparable, but distinct, SL4 in its DRTE (described in a later section) that contains a complementary terminal loop sequence for CCCCA (red) in SL2 (**Fig 2C**). These comparative observations support the existence and proposed relevance of PEMV1’s SL2 in mediating the initial union of PRTE and DRTE via a SL2/SL4 kissing-loop interaction.

### Functional analysis of the CCCC/GGGG (red) interaction in PEMV1 sg mRNA

**Fig 3:**
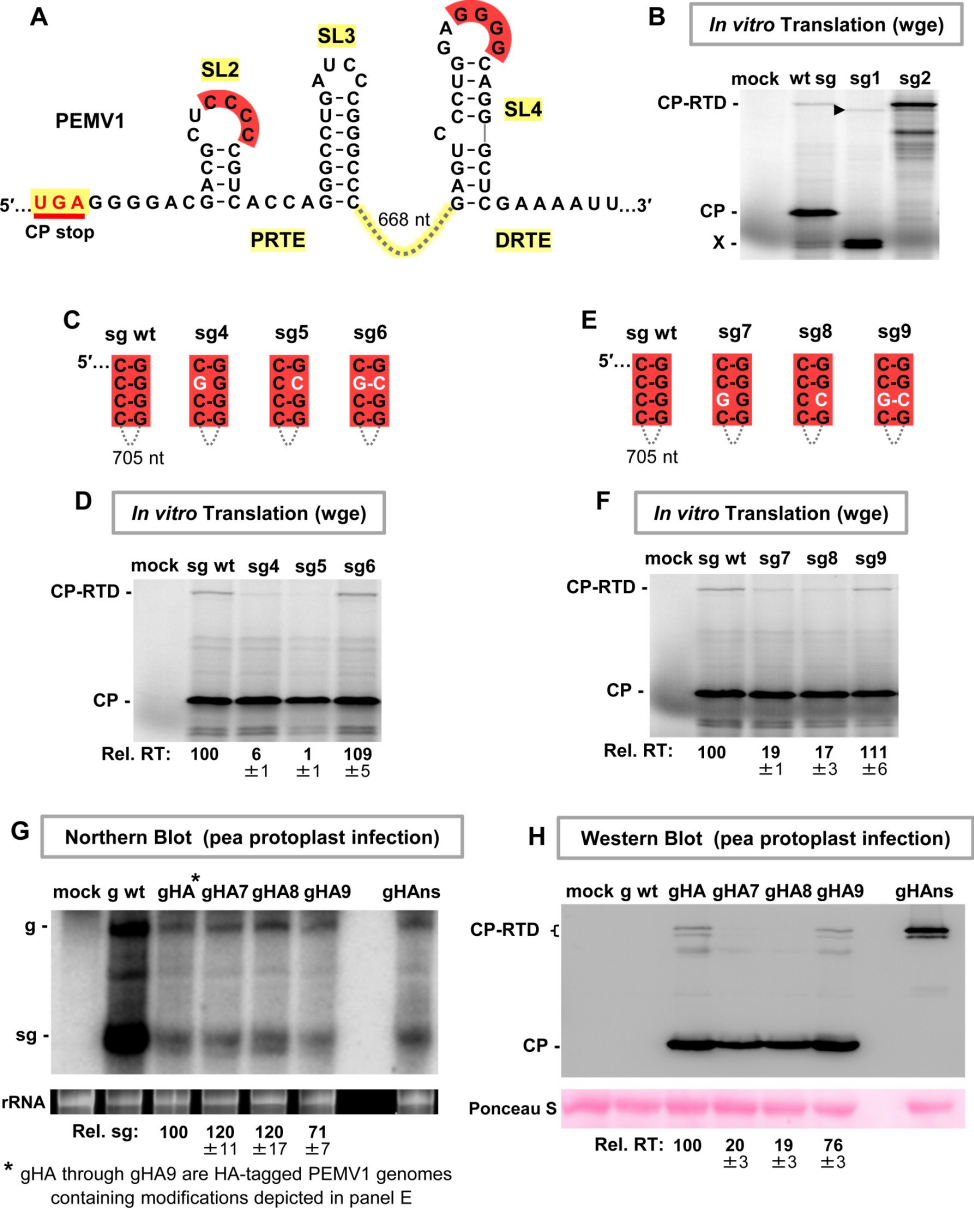
Assessing the red long-distance RNA-RNA interaction. **(A)** Secondary structures for the PRTE alternative fold and DRTE in PEMV1. The intervening 668 nucleotides between SL3 and SL4 are depicted by a connecting dashed line. **(B)** Wheat germ extract (wge) in vitro translation assay testing wt and mutant PEMV1 sg mRNAs. In mutants sg1 and sg2, the CP start codon and the CP stop codon, respectively, were inactivated (AUG → CAG and UGA → GGA). The sg mRNAs tested are indicted above each lane and the identities of the translated viral proteins are indicated on the left. The X-designated doublet band likely represents translation initiation at internal start codons in the CP ORF, and their probable readthrough products are indicated by the arrowhead. **(C)** and **(E)** Compensatory mutations introduced in the sg mRNA to test the red interaction. Nucleotide substitutions are shown in white. **(D)** and **(F)** In vitro translation analyses of the sg mRNAs shown in panels C and E, respectively. Average relative readthrough (Rel. RT) levels (±SE) calculated from three independent trials are shown below each lane. **(G)** Northern blot analysis of total nucleic acids isolated from pea protoplasts transfected with wt and HA-tagged mutant PEMV1 genomes. gHA, gHA7, gHA8, gHA9 and gHAns each contain a triple HA tag inserted 6 amino acids from the CP N-terminus. Tagged genomic mutants gHA7, gHA8 and gHA9 contain the same compensatory mutations as shown in panel E, and genomic mutant gHAns has the same CP stop codon knockout substitution as mutant sg2 in panel B. Substitutions in the DRTE in gHA8 and gHA9 lead to an arginine to serine amino acid change in CP-RTD. Positions of the genome (g) and sg mRNA (sg) are shown on the left side of the blot. Average sg levels (±SE) were calculated from three independent trials and are displayed below each lane. An ethidium bromide-stained rRNA loading control is shown below the Northern blot. **(G)** Western blot analysis of total proteins extracted from the same pea protoplast infections as in panel G. Identities of the detected viral proteins are indicated on the left and averaged Rel. RT levels (±SE) from three independent trials are shown under each lane. Ponceau S-stained loading control of the blot is shown below.

A wheat germ extract (wge) in vitro translation system was employed to assess modulation of PEMV1 CP readthrough by the identified RNA elements, starting with the red partner sequences (**Fig 3A**). To accurately assign the identity of translational products, the wt sg mRNA, a sg mRNA with the CP start codon inactivated by changing AUG to CAG (mutant sg1), and a sg mRNA with the UGA CP stop codon altered to glycine-coding GGA (mutant sg2) were tested. The results showed that both CP and CP-RTD were produced from wt sg mRNA, as confirmed by the absence of the former in the CP AUG knockout (sg1) and the increased levels of the latter in the CP UGA knockout (sg2) (**Fig 3B**). Two smaller minor products were also generated from wt sg mRNA (**Fig 3B, denoted by X**). These bands likely represent translational initiation at inframe downstream AUGs in the CP open reading frame (ORF), because in mutant sg1 (CP AUG knockout) their accumulation increased and a corresponding smaller readthrough product(s), denoted by an arrowhead, appeared (**Fig 3B**).

Having established that the wge system yielded readily detectable amounts of CP-RTD, the proposed red CCCC/GGGG interaction between SL2 and SL4 was investigated (**Fig 3A**). Sets of compensatory substitutions were introduced individually at two different nucleotide positions in the complementary red sequences (**Fig 3C and 3E**). In vitro translation analysis of corresponding wt and mutant sg mRNAs showed that the relative readthrough levels correlated with base-pairing capacity between the terminal loop sequences (**Fig 3D and 3F**). That is, when base pairing was disrupted, relative readthrough levels dropped below 20% that of wt (**Fig 3D, mutants sg4 and sg5; Fig 3F, mutants sg7 and sg8**), while restoration of base pairing in compensatory mutants sg6 and sg9 rescued relative readthrough to wt levels (**Fig 3D and 3F**).

To determine if the results obtained from in vitro translation assays reflected activity in corresponding in vivo viral infections, an N-terminal triple-HA tag was introduced into the CP ORF in the full-length PEMV1 genome (creating gHA), thus allowing for immunological detection of CP and CP-RTD. The same red sequence-targeting mutations in sg mRNA mutants sg7, sg8 and sg9 (**Fig 3E**) were then introduced into the gHA genomic context, creating gHA7, gHA8, and gHA9, and the tagged viral genomes were transfected into pea protoplasts. Infections also included gHAns as a control, which was a mutant genome in which the CP stop codon was converted to a glycine sense codon (UGA → GGA). Northern blot analysis revealed that HA-tagged genomes and sg mRNAs accumulated to lower levels than their wt counterpart (**Fig 3G**), likely due to the tag interfering with virus packaging and/or other intracellular viral processes. However, the accumulation levels of the sg mRNAs in the tagged virus infections were reasonably comparable, and examination of corresponding relative readthrough levels revealed results that were consistent with those from in vitro assays (**compare Fig 3H and 3F**). Combined, these in vitro and in vivo findings provide compelling evidence for the requirement of the red CCCC/GGGG interaction for optimal readthrough and validated use of the wge system for further analysis.

### Additional RNA elements are required for efficient readthrough

**Fig 4:**
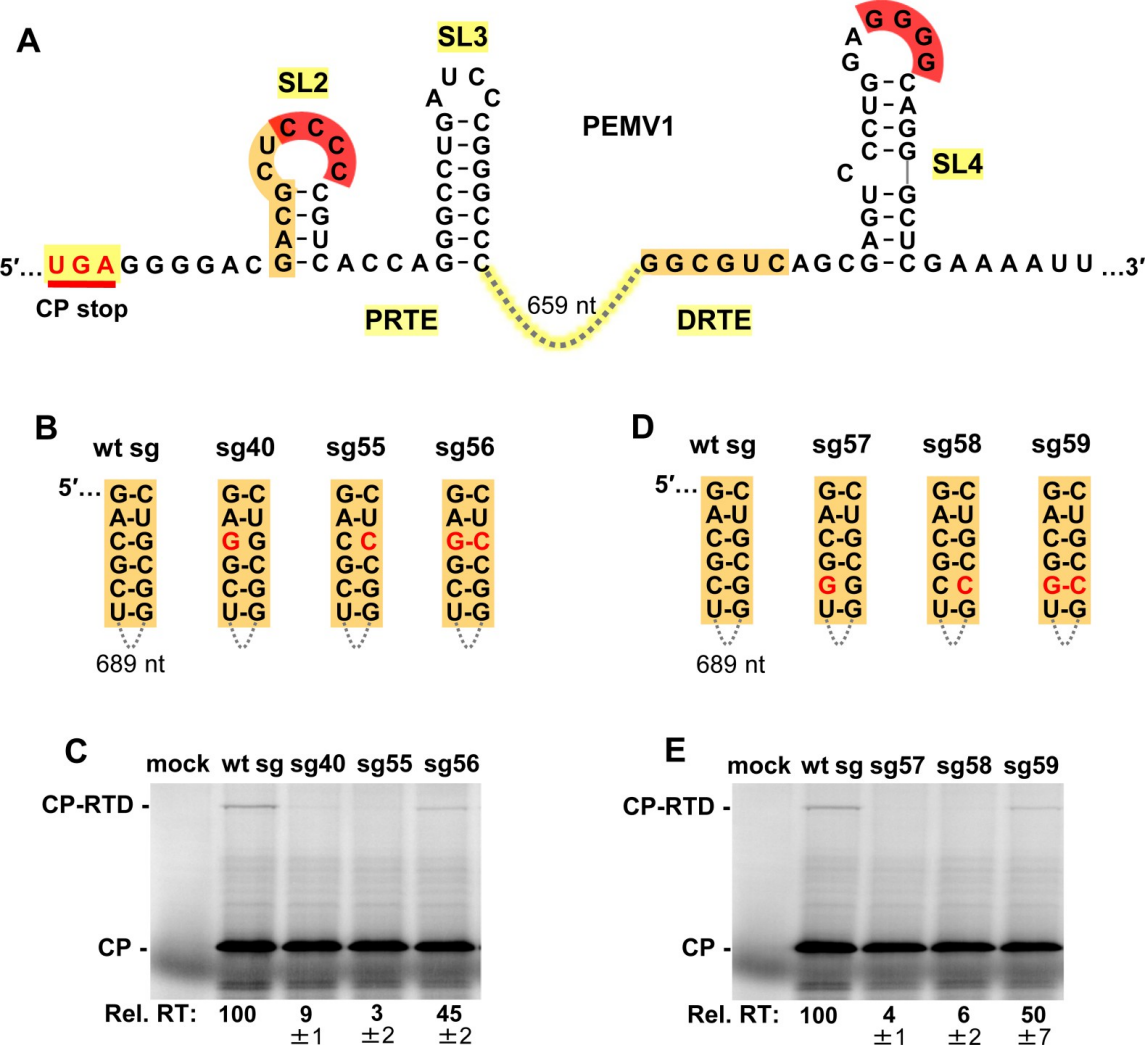
Assessing the orange long-distance RNA-RNA interaction. **(A)** RNA secondary structures of PRTE and DRTE in PEMV1. **(B)** and **(D)** Compensatory mutations introduced in the sg mRNA to test the orange interaction. Nucleotide substitutions are shown in red. **(C)** and **(E)** In vitro translation analyses of the wt and mutant sg mRNAs shown in panels B and D, respectively. Averaged Rel. RT levels (±SE) collected from three independent trials are shown below each lane.

Formation of the long-distance red CCCC/GGGG interaction would position the identified complementary orange sequences in the PRTE and DRTE in close proximity (**Fig 4A**). In vitro translation of compensatory mutants targeting two different base pairs in the orange partner sequences in the sg mRNA (**Fig 4B and 4D**) supported functional base-pairing (**Fig 4C and 4E**). Notably, in both cases, only partial rescue of readthrough (~45–50% of wt) was observed for compensatory mutants sg56 and sg59 (**Fig 4C and 4E**). This lower level of rescue could be related to the substitutions in the orange sequence in the PRTE interfering with presentation of the red CCCC in the terminal loop of SL2, because the orange sequence forms the 5′ half of the stem in SL2 (**Fig 4A**). Regardless, the obtained results support an important role for the orange interaction in promoting readthrough efficiency.

**Fig 5:**
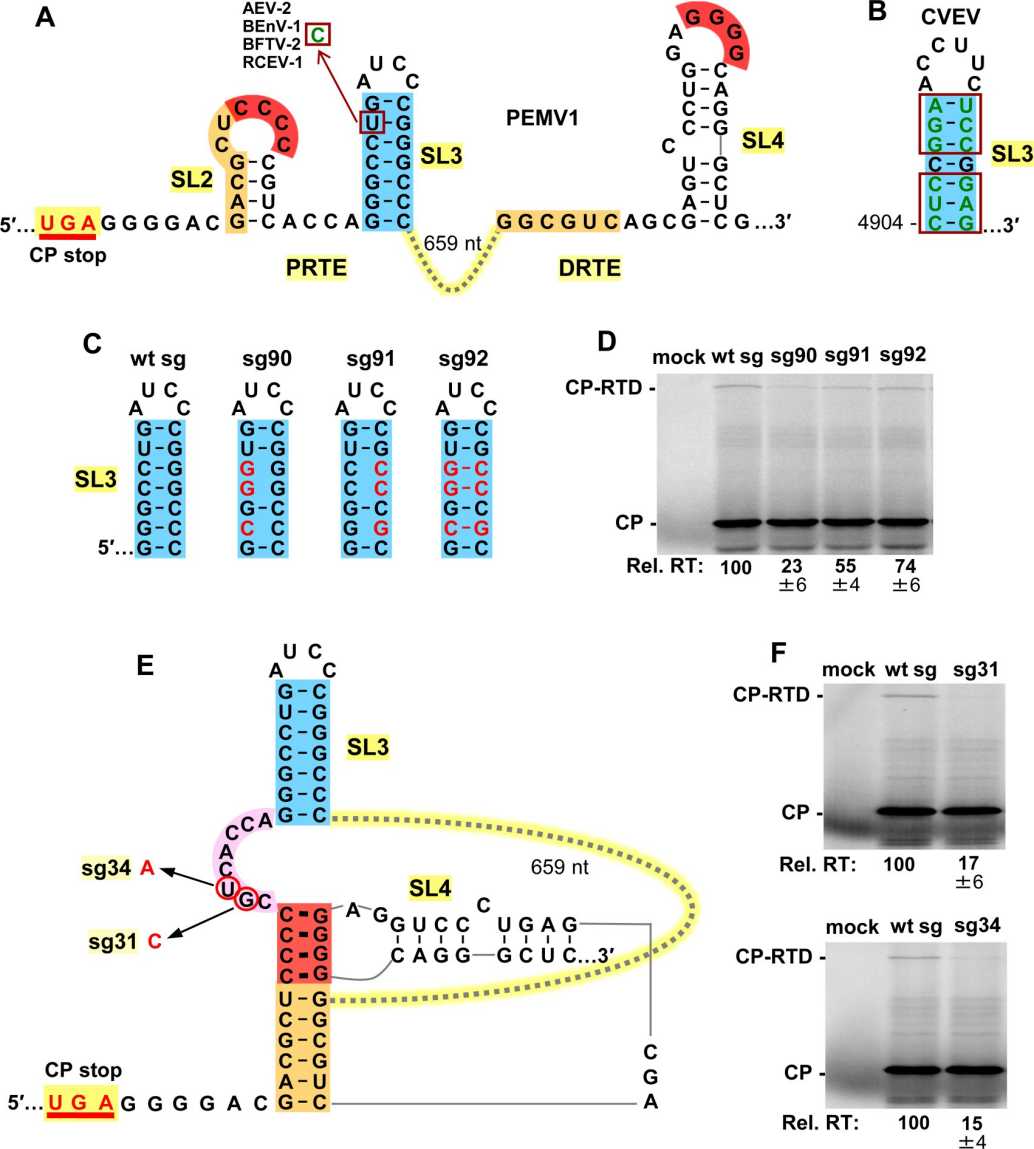
Assessing the local SL3 (blue) in the PRTE. **(A)** RNA secondary structures of PRTE and DRTE in PEMV1. A nucleotide mono-variation (U to C) in the SL3 of four enamoviruses that maintains base pairing is shown (boxed). Alfalfa enamovirus-2 (AEV-2, KY985463.1), bean enamovirus-1 (BEnV-1, MZ361924), birdsfoot trefoil virus-2 (BFTV-2, NC_048296) and red clover enamovirus-1 (RCEV-1, MN412742). **(B)** SL3 of citrus vein enation virus (CVEV, NC_021564). Covariations in the SL3 stem that maintain pairing are boxed. **(C)** Compensatory mutations introduced into SL3, with substitutions depicted in red. **(D)** Results of in vitro translation reactions for the sg mRNAs shown in panel C. Averaged Rel. RT levels (±SE) calculated from three independent trials are shown below each lane. **(E)** Proposed RNA secondary structure when the red and orange long-distance interactions between the PRTE and DRTE occur. The linker sequence between red and blue helices is highlighted in pink, with corresponding substitutions in mutant sg mRNAs circled and indicated in red. **(F)** Results of in vitro translation reactions for mutant sg mRNAs shown in panel E. Averaged Rel. RT levels (±SE) calculated from three independent trials are shown below each lane.

The importance of SL3 (blue), localized within the PRTE region, was also assessed due to its proximity to the other functionally relevant PRTE sequences (i.e. orange and red) and its conservation among enamoviruses (**Fig 5A**). Regarding the latter point, four enamoviruses contain a U-to-C substitution in the stem of their SL3s that maintains pairing (**Fig 5A**), while CVEV contains a SL3 with multiple covariant base pairs (**Fig 5B, boxes**). Compensatory mutations in sg mRNAs were designed to simultaneously target three base pairs in the GC-rich stem of PEMV’s SL3 (**Fig 5C**) and results from wge assays indicated that stability of the stem contributes to CP stop codon readthrough (**Fig 5D**), albeit to a lesser degree than the associated long-distance interactions.

Formation of the two long-distance RNA-RNA interactions (red and orange) between the PRTE and DRTE would lead to an RNA structure with a large intervening sequence (659 nt) (**Fig 5E**). In this structure, the red helix would likely coaxially stack on the orange helix below, with the red helix separated from SL3 (blue) by an 8 nt long intervening sequence (pink) (**Fig 5E**). The location of this small linker sequence between two functionally important structures suggested that it too could be important for readthrough. Consequently, two separate single nucleotide substitutions were introduced into the intervening pink sequence (**Fig 5E**). Results from translational assays revealed that both substitutions had notable detrimental effects on relative readthrough levels (**Fig 5F**), confirming an important role for the pink linker sequence.

### A third long-distance RNA-RNA interaction is required for readthrough

**Fig 6:**
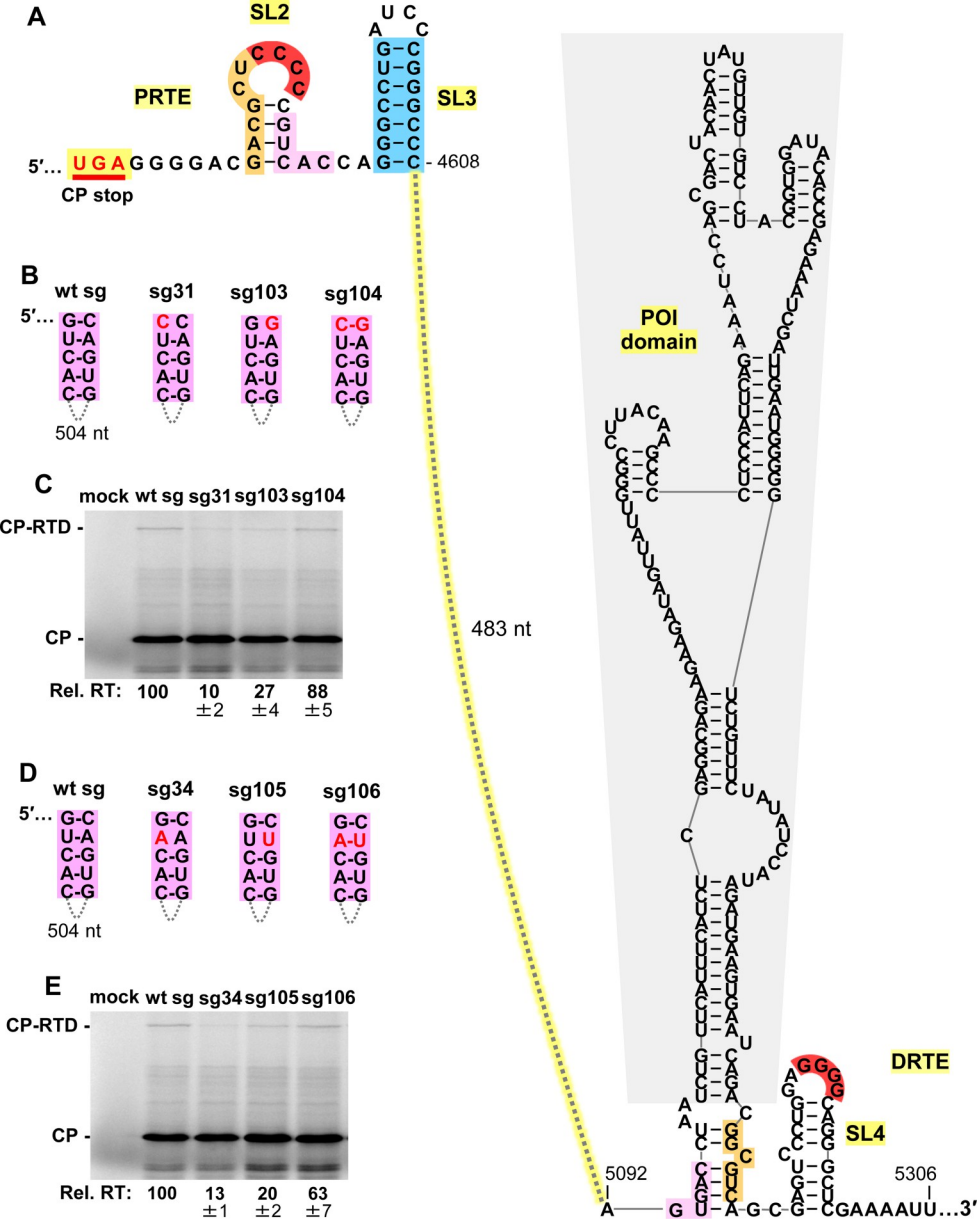
Assessing the pink long-distance RNA-RNA interaction. **(A)** RNA secondary structures of PRTE and DRTE in PEMV1, including the POI domain (grey shading). The complementary sequences highlighted in pink represent a third long-distance RNA-RNA interaction between PRTE and DRTE. **(B)** and **(D)** Compensatory mutations introduced in the sg mRNA context to test the pink interaction. Nucleotide substitutions are shown in red. **(C)** and **(E)** In vitro translation analyses of the wt and mutant sg mRNAs shown in panels B and D, respectively. Averaged Rel. RT levels (±SE) collected from three independent trials are shown below each lane.

Like the orange and red sequences in the PRTE, we reasoned that the pink sequence (**Fig 6A, top left**) could also function by pairing with a complementary sequence. Potential base-pairing partner sequences for the PRTE’s pink segment were initially sought close to the red and orange sequences in the DRTE. Although complementary sequences were identified nearby, none proved to be functionally relevant. A continued search ultimately identified a partially complementary 5 nt long sequence (pink) located some 170 nucleotides upstream from the orange segment in the DRTE (**Fig 6A, bottom right**). Compensatory mutagenesis of two different base pairs followed by translational analyses revealed a critical role for the pink PRTE-DRTE long-distance interaction in facilitating optimal CP readthrough (**Fig 6B–6E**). As with the orange interaction (**Fig 4**), the inability to recover full activity with restored pink pairing may be related to concurrent destabilization of the stem of SL2 and reduced presentation of the red CCCC (**Fig 6A**). Notably, although the 5 nt long pink sequence is located 170 nucleotides upstream from the orange and red in the DRTE, the intervening 170 nucleotides are predicted, in the context of the full-length wt sg mRNA (**Fig 1C**), to fold into a small RNA domain, herein termed the pink-orange intervening (POI) domain (**Fig 6A**, **grey shading**). Formation of the POI domain would colocalize the red, orange, and pink sub-elements of the DRTE (**Fig 6A, bottom right**), thereby facilitating their simultaneous interaction with their corresponding localized partner sequences in the PRTE. Collectively, the results show that optimal PEMV1 CP stop codon readthrough depends on three long-distance RNA-RNA interactions (red, orange, and pink) and a local stem-loop structure, SL3 (blue).

### Role of DRTE’s SL4 and a potential fourth long-distance interaction

**Fig 7:**
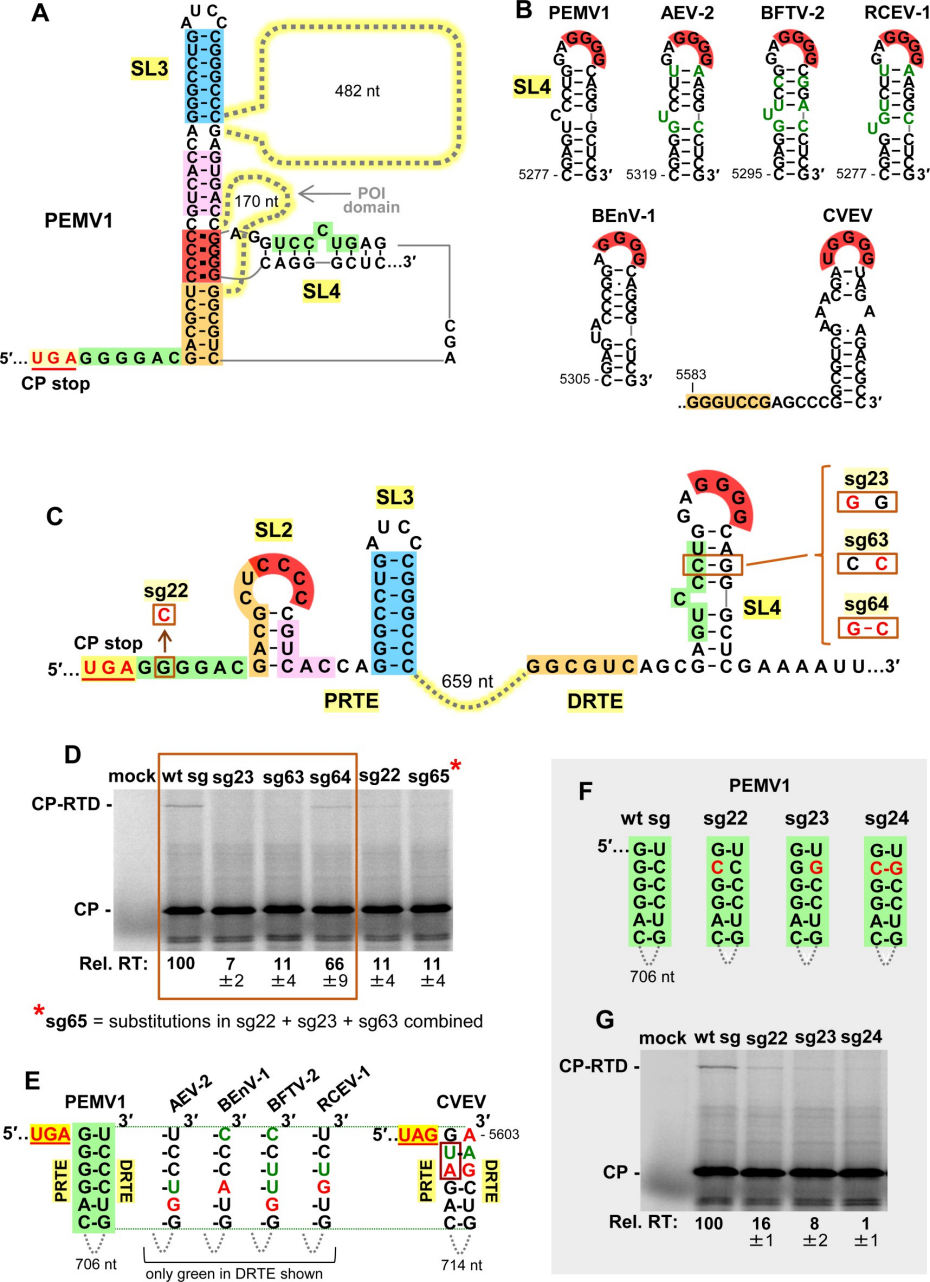
Assessing SL4 and a potential fourth PRTE/DRTE interaction. **(A)** RNA secondary structure model for the readthrough structure when red, orange, and pink complementary sequences are paired. **(B)** Conservation of SL4 in DRTEs among the members of the genus Enamovirus: PEMV1, AEV-2, BFTV-2, RCEV-1, BEnV-1, and CVEV. In the top row, mono- and co-variations that maintain base pairing of SL4 are shown in green. **(C)** RNA secondary structures of PRTE and DRTE in PEMV1, with substitutions targeting the green PRTE sequence and the stem of SL4 boxed and shown in red. Segments in the putative fourth PRTE/DRTE long-distance interaction are highlighted in green. **(D)** In vitro translation analyses of the wt and mutant sg mRNAs. The boxed area represents results from the SL4 stem compensatory mutants shown to the right in panel C. Averaged Rel. RT levels (±SE) collected from three independent trials are shown below each lane. (**E)** Conservation of the green pairing between the PRTE and DRTE among enamoviruses. The green PRTE sequence is 100% conserved (except for CVEV), while complementary green DRTE sequences have variations that maintain (green) or potentially destabilize (red) the base-pairing of the green partner sequences. **(F)** Compensatory mutations introduced in PEMV1 sg mRNA that target the long-distance PRTE/DRTE green base-pairing interaction. **(G)** In vitro translation analyses of the wt and mutant sg mRNAs shown in panel F. Averaged Rel. RT levels (±SE) collected from three independent trials are shown below each lane.

Simultaneous base pairing between complementary red, orange, and pink sequences would collectively lead to the assembly of an extended quasi-continuous helix (**Fig 7A**), with the 170 nt long POI domain and a larger 482 nt long domain extending from the helical intersections. The junctions of the adjacent helices are likely stabilized via coaxial stacking, which for the blue-pink and pink-red helical joints could involve non-canonical base pairs forming above (AG, CA) and below (CC) the pink helix (**Fig 7A**). In this structure, SL4’s stem could, as shown, remain paired while its loop interacts with its red partner sequence in the PRTE (**Fig 7A**). However, an alternative long-distance interaction (green) was noted in which the 5′-portion of SL4’s stem could base-pair with a 6 nt complementary sequence immediately downstream from the CP stop codon (**Fig 7A, green**). Thus, the stem sequence in SL4 could first function locally in the DRTE to present the GGGG (red) sequence and subsequently participate in a fourth PRTE/DRTE (green) interaction. SL4’s role in presenting GGGG (red) is strongly supported by comparative structural analysis among enamoviruses, which revealed covariation within the stem (alfalfa enamovirus-2, AEV-2; bird’s-foot trefoil enamovirus, BFTV-2; and red clover enamovirus-1, RCEV-1) and alternative SL4 folds (Bean enamovirus-1, BEnV-1 and citrus vein enation virus, CVEV) (**Fig 7B**). Additionally, the analysis of PEMV1 sg mRNAs with compensatory mutations in the stem of SL4 (**Fig 7C**, **right**) confirmed the importance of pairing in its stem (**Fig 7D, boxed lanes**).

Comparative structural analysis of the potential long-distance green interaction revealed that for most enamoviruses (except CVEV) the green sequence in the PRTE is strictly conserved, with substitutions in partner green sequences in their DRTEs that generally maintained complementarity (nucleotides in green), or generated non-canonical GA or AG pairs (nucleotides in red) (**Fig 7E**). CVEV’s green sequence in its PRTE contains two substitutions (boxed) compared to that of the other enamoviruses (**Fig 7E**), and collectively maintains a potential green interaction that could include GA and AG pairs [36–38]. Accordingly, the structural comparisons suggest the possibility of a fourth functionally relevant long-distance green PRTE/DRTE interaction. Indeed, if sterically feasible, the green interaction would extend the quasi-continuous helix at the base and presumably further enhance the structure’s stability (**Fig 7A**). To address this possibility, compensatory mutations were introduced into the green partner sequences in sg mRNAs and tested in translational assays (**Fig 7F and 7G**). Disruptive mutants (sg22 and sg23) notably decreased readthrough, while the restorative mutant (sg24) caused further reduction (**Fig 7G**). Moreover, combining the green interaction-restoring changes in mutant sg24 with an additional substitution (**Fig 7C, sg63**) that simultaneously restored pairing in the stem of SL4 (thereby generating sg65) did not lead to recovery of readthrough (**Fig 7D**). Therefore, nucleotide identity within the PRTE’s green sequence is important, but its role may be independent of pairing with the DRTE’s complementary green sequence. Accordingly, while our results corroborate an important role for the stem of SL4 in presenting the red GGGG partner sequence in the DRTE, they do not support, but also do not conclusively preclude, its involvement in a fourth long-distance green PRTE/DRTE interaction.

### PLRV readthrough signal involves a long-distance RNA-RNA interaction

**Fig 8:**
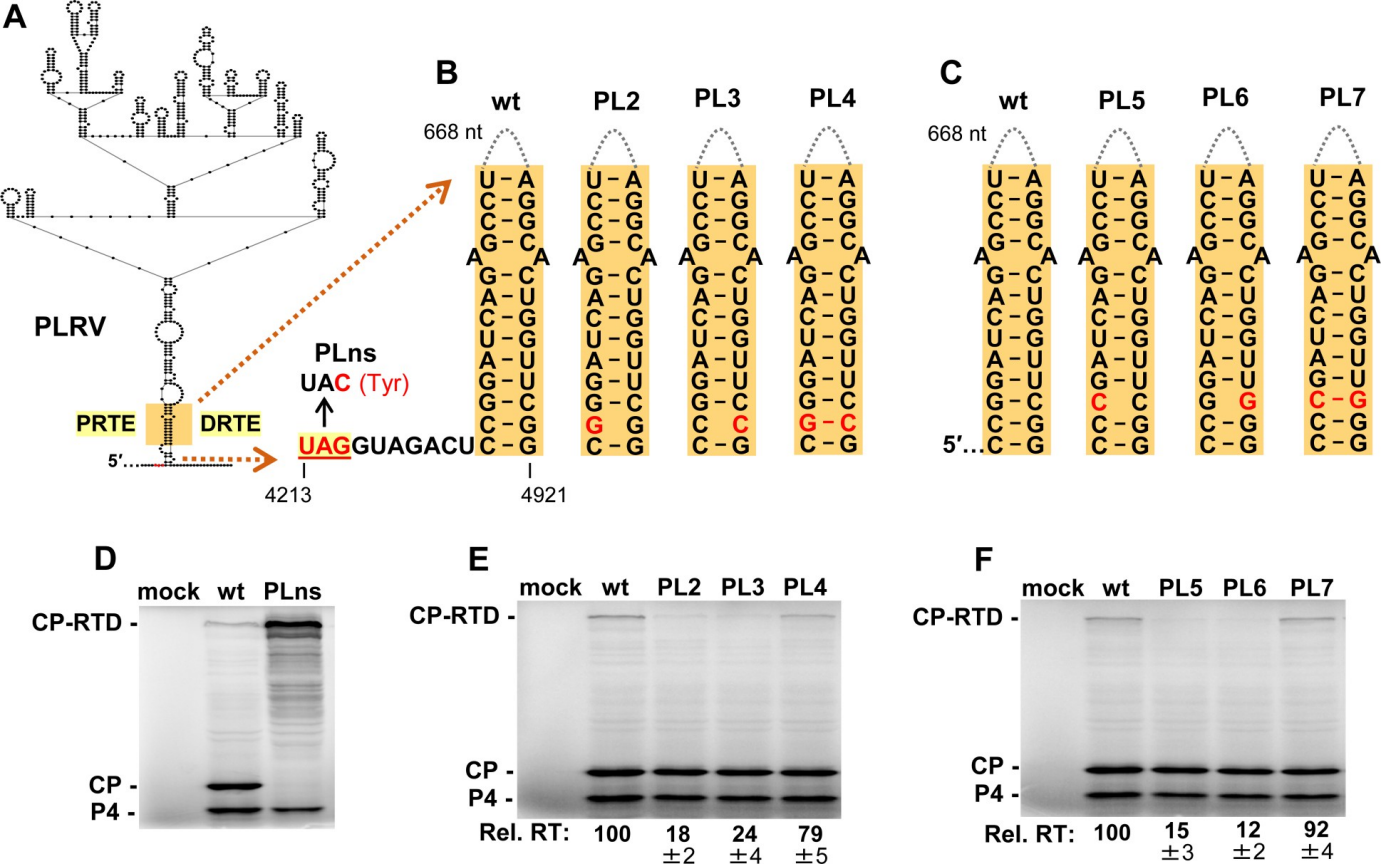
Assessing the PRTE/DRTE interaction in PLRV. **(A)** RNA secondary structure model for a central region of the PLRV sg mRNA based on *RNAStructure* [34] and rendered using *RNA2Drawer* [57]. In the structure, small black circles represent nucleotides, with those corresponding to the PLRV CP UAG stop codon shown in red. Highlighted in orange are the PRTE and DRTE sequences previously proposed to base-pair and regulate CP readthrough in PLRV [13]. **(B)** and **(C)** Compensatory mutations introduced in PLRV sg mRNA that target the orange PRTE/DRTE base-pairing interaction, and mutant PLns, in which the CP stop codon was inactivated. **(D)**, **(E)** and **(F)** In vitro translation analyses of the wt and mutant PLRV sg mRNAs shown in panels B and C. Averaged Rel. RT levels (±SE) collected from three independent trials are shown below each lane.

A previous study identified a PRTE and DRTE in PLRV that were both shown to be essential for efficient CP-RTD production [13]. Although these sequences exhibited notable complementarity, efforts to experimentally demonstrate a PRTE/DRTE pairing requirement for readthrough were unsuccessful [13]. Due to PLRV’s close relationship to PEMV1, we sought to assess the necessity for such pairing and deduce the RNA structure formed. Initially, the secondary structure of wt PLRV sg mRNA was modeled using the *RNAStructure* folding program [34]. Interestingly, within the full-length sg mRNA fold, the previously identified PRTE and DRTE sequences (orange) were predicted to be paired to each other at the base of a large RNA domain (**Fig 8A**). In the prior attempt to generate informative sg mRNA compensatory mutants, several nucleotides were targeted simultaneously for substitution [13]. We reasoned that this approach likely hindered important local folding in one or both regions and/or the modified PRTE or DRTE inadvertently bound to non-cognate partner sequences elsewhere in the sg mRNA. We therefore designed our compensatory mutations as single nucleotide changes that would disrupt the bottom of the proposed structure while minimally altering the partner sequences. This strategy would both destabilize the overall structure and alter the functionally important distance between the UAG and the base of the readthrough-promoting structure (**Fig 8B and 8C**). Also, contrary to prior reports [13,39], we were able to detect synthesis of a PLRV CP-RTD product using wge assays, as confirmed by its level increasing upon knockout of the CP stop codon in sg mRNA mutant PLns (**Fig 8D**). Using the wge system to test wt and mutant PLRV sg mRNAs, we observed that both sets of compensatory mutants yielded results consistent with base pairing of the orange sequences in the PRTE and DRTE being required for optimal CP-RTD production (**Fig 8E and 8F**). These results demonstrate that the previously proposed long-distance interaction in PLRV [13] is indeed essential for optimal readthrough of its CP stop codon.

## Discussion

Survival of PEMV1 and PLRV depends on aphid-mediated host-to-host transmission, which is conferred by their CP-RTD minor capsid proteins generated via programmed ribosome readthrough [40]. In this study we performed a detailed investigation of the regulation of CP-RTD production in PEMV1 and developed an elaborate multi-helix model for the readthrough structure. In contrast, our assessment of the PLRV readthrough signal indicated a simple single-helix RNA structure. Below, different readthrough structures are discussed, the PEMV1 and PLRV readthrough structures are compared, and hypothetical models for the assembly of PEMV1 and PLRV readthrough signals are proposed.

### Long-distance readthrough structures in other viruses

**Fig 9:**
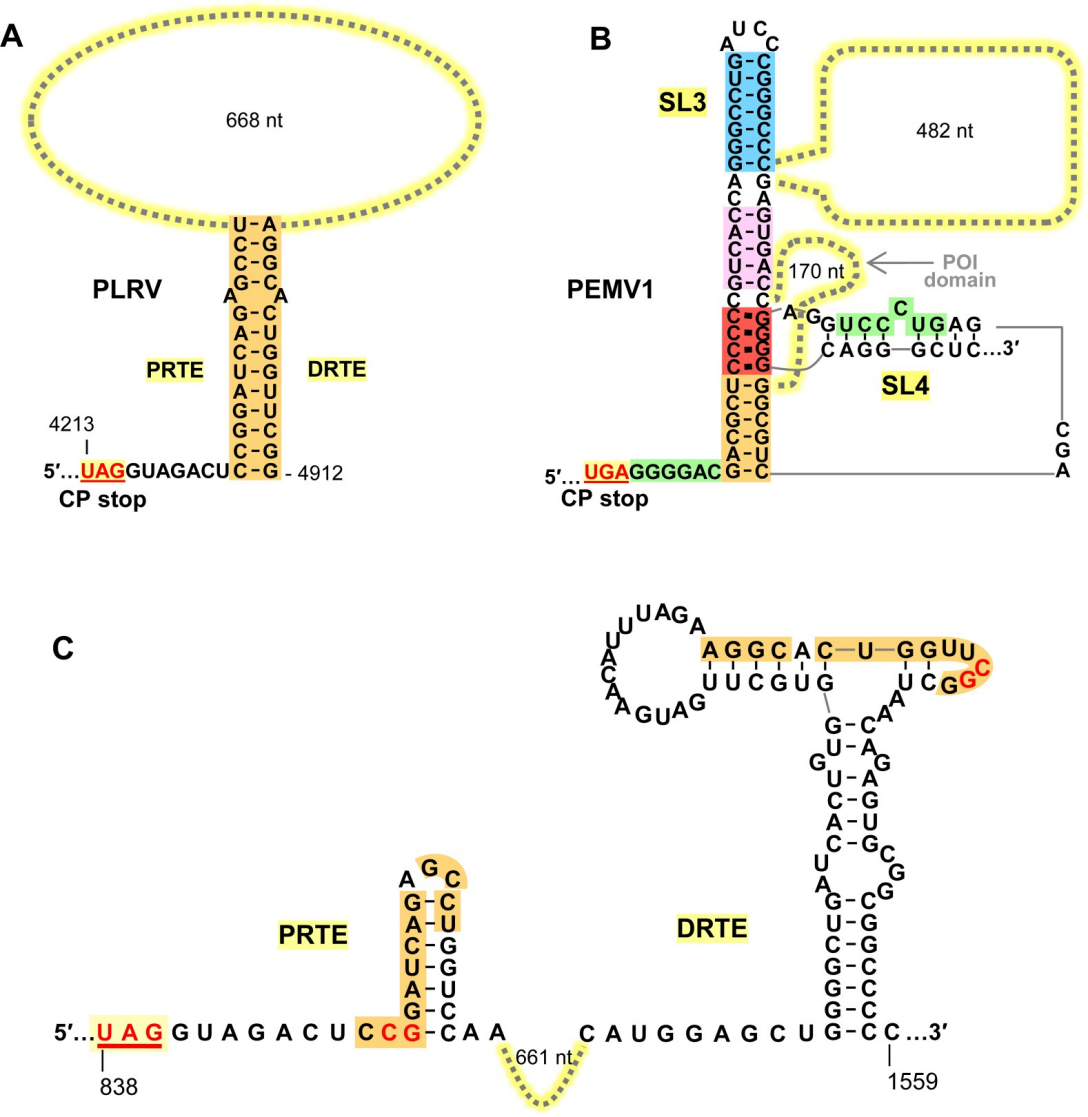
Comparison of PLRV and PEMV1 readthrough structures. **(A)** Predicted readthrough structure of PLRV showing the key orange PRTE/DRTE interaction. **(B)** Proposed readthrough structure for PEMV1, including local and long-distance interactions. **(C)** Predicted local RNA secondary structures for PRTE and DRTE [13] regions in PLRV. Nucleotides shown in red were targeted for compensatory mutational analysis in Fig 8.

Programmed stop codon readthrough is commonly used by RNA viruses to produce their RdRps or minor CPs [1]. In some cases, readthrough stimulating signals are localized immediately downstream from corresponding stop codons. Murine leukemia retrovirus relies on a compact RNA pseudoknot structure situated 8-nt downstream from its gag stop codon for pol translation [41], while in tobacco mosaic virus a 6 nt-long linear sequence directly after the stop codon promotes readthrough production of its RdRp [9]. In other viruses, bipartite readthrough signals, separated by intervening sequences, are employed. For example, alphaviruses utilize a simple helical readthrough structure, similar to that in PLRV (**Fig 9A**), for production of their RdRps [5]. However, although comparable with respect to their basic stem structures, the intervening sequences in alphaviruses are considerably shorter than that in PLRV (i.e. ~100–150 nt versus ~670 nt, respectively).

Arguably the best studied viruses employing long-distance interactions for readthrough are genera in the family Tombusviridae (Tombusvirus, Betanecrovirus and alphacarmovirus), all of which use long-distance RNA-RNA base pairing (spanning kilobases) to mediate readthrough expression of their RdRps [6–8]. In contrast to readthrough in the sg mRNAs in PEMV1 and PLRV, readthrough in tombusvirids occurs in the full-length viral genomes, and with corresponding DRTEs located in their genomic 3′UTRs. This placement coincides with genomic replication elements, allowing for potential crosstalk between the two processes. For instance, the DRTE of the tombusvirus carnation Italian ringspot virus is integrated with a genome replication element in the genomic 3′UTR and, importantly, the functional structures of the DRTE and replication element are mutually-exclusive RNA conformations. This overlapping arrangement acts as an RNA switch that dictates whether genomic minus-strand synthesis or translational readthrough proceeds, thereby coordinating these two opposing processes [6]. In contrast, the DRTEs for PEMV1 and PLRV are positioned centrally in the coding regions of their RTDs (**Fig 1B**). Thus, although possible, the remote locations of these DRTEs are likely not related to regulation of other sg mRNA processes. Instead, their positions are more likely the consequence of random but productive (for readthrough) initial long-distance interactions, which were maintained and further optimized.

The PRTEs of some tombusvirids can assume alternate structures or have flexible adjacent structures important for readthrough efficiency. The PRTE of the alphacarmovirus turnip crinkle virus can adopt two alternative structures, one which is nonfunctional and the other that is functional [8]. In tobacco necrosis betanecrovirus, a downstream PRTE-adjacent structure that influences readthrough efficiency has both active and inactive conformations [42]. Alternative RNA conformations such as these provide additional avenues for regulating readthrough, and illustrate the importance of considering local context and structural flexibility when investigating regulatory RNA elements. Indeed, as alluded to earlier (**Fig 2A**), alternative local conformations are also likely relevant in PEMV1’s PRTE.

For both betanecroviruses and tombusviruses, in addition to their PRTE/DRTE interactions, efficient RdRp readthrough expression requires an extra long-distance RNA-RNA interaction, termed the upstream linker/downstream linker (UL/DL) interaction [6,43], which is also essential for viral genome replication [44]. Accordingly, these viruses employ two distinct long-distance interactions for readthrough, one involved in forming the readthrough structure (PRTE/DRTE) and another that serves an essential accessory role (UL/DL). Since the UL/DL interactions reside within the ~3 kb intervening sequence between the PRTE and DRTE partner sequences, it was proposed that they likely function to help unite the PRTE and DRTE [6,43]. In this regard, the possibility of intervening sequence assisting in the formation of the PRTE/DRTE interaction in PLRV is discussed in the next section.

### The PEMV1 readthrough structure versus PLRV’s

Enamovirus, Luteovirus, and Polerovirus genera are related based on amino acid conservation of their CP and CP-RTD [32,45]. Members of these genera are also predicted to contain bipartite readthrough regulatory signals separated by ~600 to ~800 nt [12,13]. Notably, they all have the same relative positioning of their PRTEs and DRTEs in the CP-RTD coding region [13]. This suggests that CP/CP-RTD coding and associated readthrough signal were adopted by an enamo/polerovirus common ancestor prior to its divergence into two distinct genera, while a recombination event introduced the 3′-proximal structural gene cassette into luteoviruses, which contain tombus-like polymerases [46–48]. Despite their distinct evolutionary histories, these genera have maintained commonalities in their strategies for mediating readthrough.

Of the three genera, poleroviruses and enamoviruses are most similar [16]. Yet a comparison of the prototype species, PLRV and PEMV1, revealed clear differences in their approach to inducing readthrough (**Fig 9A and 9B**). PLRV’s CP ORF and those of all known poleroviruses terminate with an UAG stop codon, while PEMV1 and all known enamoviruses (except for CVEV) use UGA. Proteomic analysis of PLRV’s CP-RTD revealed that the UAG is decoded ~89% of the time by tRNA^Gln^ [13]. The tRNA responsible for decoding PEMV1’s UGA is currently unknown. Corresponding PRTEs and DRTEs in PLRV and PEMV do not share any noteworthy sequence identity (**Fig 9A and 9B**). Dissimilarity also extends to the predicted local RNA secondary structures at these two locations. For PLRV, prior solution structure probing and mutational analyses [13] determined that the orange DRTE sequence involved in forming the readthrough structure resides in a local stem-loop structure, with most of the orange nucleotides paired (**Fig 9C, right**). The local structure in the PRTE region was not investigated [13], but thermodynamic predictions suggest that this segment likely includes a small RNA stem-loop that sequesters most of the PRTE’s orange sequence (**Fig 9C, left**). Based on these predictions, the PRTE and DRTE regions do not adopt conformations that would effectively nucleate PLRV’s orange interaction. This suggests that PLRV uses a different strategy for uniting these sequences, and secondary structure predictions of the full-length PLRV sg mRNA indicate that this could be accomplished through global folding, where PRTE and DRTE form the closing ends of a large RNA domain (**Fig 8A**). That is, the folding of subdomains within the large domain would act to bring the partner sequences together. In contrast, folding predictions for PEMV1 sg mRNA indicate that the PRTE and DRTE are located in different RNA domains (**Fig 1C**), thus a unification mechanism akin to that suggested for PLRV would be less likely. Accordingly, the differences in sequence and predicted RNA structures for PLRV and PEMV1 indicate that the former likely mediates formation of its readthrough structure primarily through the folding of an independent RNA domain, while the latter initiates readthrough structure formation by stochastic nucleation of key partner sequences (i.e. red) located in different RNA domains (see next section for details).

The proposed readthrough signal for PLRV, a contiguous helix, is relatively simple (**Fig 9A**). In comparison, the PEMV1 readthrough structure is considerably more complex, consisting of a quasi-contiguous helix stabilized by coaxial stacking at stem junctions and assembled via multiple long-distance interactions involving different regions (**Fig 9B**). Though these structures differ greatly, they are both able to direct production of the requisite amounts of CP-RTD. It is intriguing that two closely related viruses have found such radically different structural solutions for readthrough. These differences are presumably the consequence of repeated sequential sampling of distinct structural variants, resulting in maintenance of those that adequately addressed functional requirements. The net result being that these viruses have evolved via divergent pathways to give rise to secondary structures of vastly contrasting complexity. Considering these extreme examples, and the predicted variability of PRTE/DRTE interactions [13], we anticipate the existence of a range of readthrough structures with different levels of complexity within the expansive and diverse polerovirus and luteovirus genera [49].

### An assembly model for PEMV1 readthrough structure

SHAPE data indicated that the default structure of the PRTE is comprised of SL1 and SL3 (**Fig 2A, left**). Importantly, although the orange sequence is predicted by SHAPE to be single stranded in the loop of SL1 (**Fig 2A**), its orange partner sequence in the DRTE is predicted to be paired (i.e. low SHAPE reactivity) and thus unavailable for pairing (**Fig 2B**). The latter interpretation is supported by the prediction that, in the context of the full-length sg mRNA, the orange sequence in the DRTE is paired with the DRTE’s pink sequence (**Fig 6A, bottom right**). Accordingly, SL1 would be limited in its ability to nucleate the PRTE/DRTE interaction via an orange pairing interaction. In the alternative PRTE fold where SL2 forms and presents the red sequence in its loop, the pink and orange sequences are paired in its stem (**Fig 6A, top left**) and thus would not be available for long-distance base-pairing with partner sequences in the DRTE; which are also predicted to be paired and unavailable (**Fig 6A, bottom right**). Consequently, the predicted local structural contexts in the alternatively-folded PRTE and the DRTE would favor the red CCCC/GGGG kissing-loop interaction, and concurrently impede the orange and pink interactions (**Fig 6A**).

**Fig 10:**
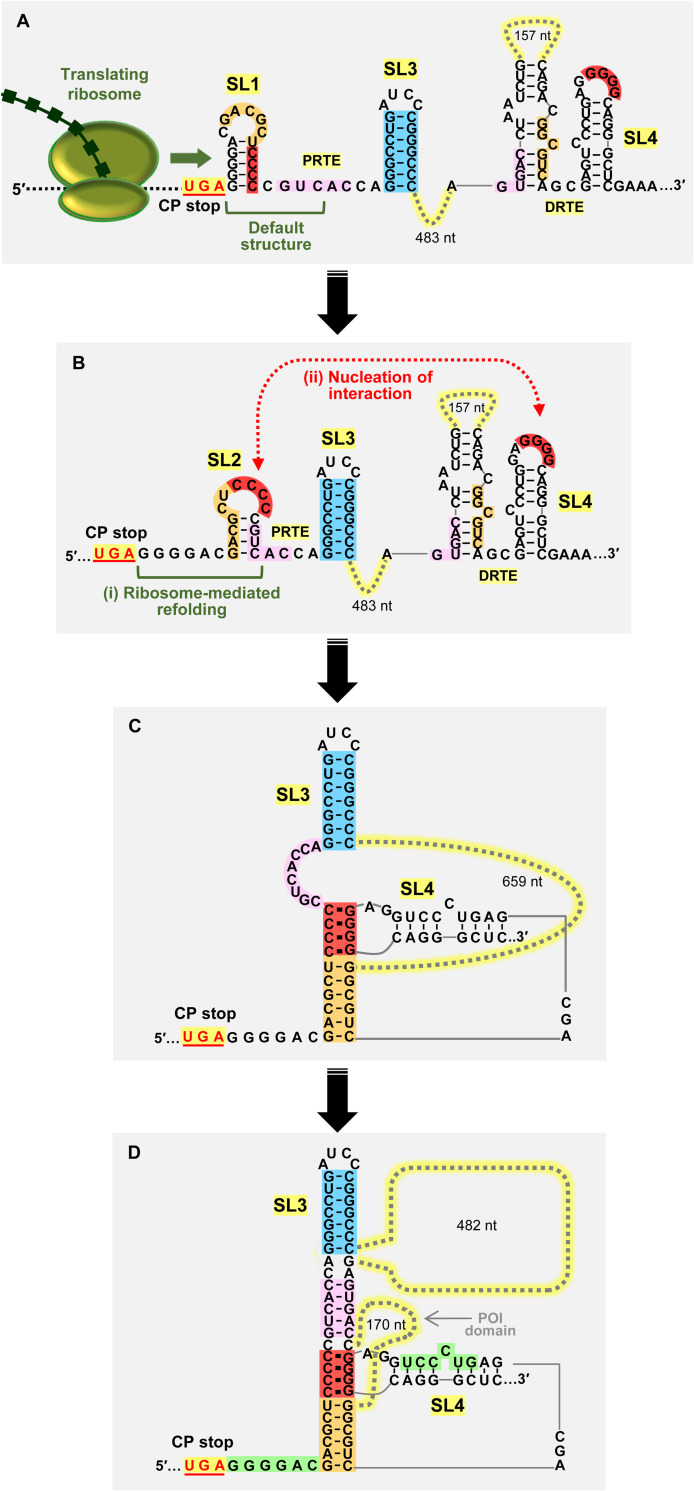
Model for assembly of the PEMV1 readthrough structure. **(A)** Default structures for PRTE and DRTE. The helicase activity of a terminating ribosome extends over SL1 and unfolds it. **(B)** (i) The sequence refolds into an alternative conformation that includes SL2. (ii) SL2 pairs with SL4 via a red sequence kissing-loop interaction and nucleates the assembly process. **(C)** Other key interactions then form, such as pairing of the orange partner sequences. **(D)** Addition of the pink interaction generates an extended helical structure, stabilized by coaxial stacking at stem junctions, that promotes efficient readthrough production of CP-RTD. A potential fourth green PRTE/DRTE interaction may also occur (not depicted), which would extend the quasi-continuous helix to the stop codon. See text for details.

Based on our experimental results, we propose a theoretical model for the assembly of the PEMV1 readthrough structure (**Fig 10**). In the PRTE, the red CCCC sequence in SL1 is initially paired in the stem of SL1 and is not available for pairing with its available red GGGG partner sequence in SL4 in the DRTE (**Fig 10A**). However, the unfolding of SL1 by the helicase activity of terminating ribosomes [50] would facilitate a SL1 to SL2 conversion (**Fig 10A**). Refolding of the PRTE sequence into the alternative CCCC-presenting SL2 (**Fig 10B, i**) would then allow for a red CCCC/GGGG kissing loop interaction with SL4 in the DRTE (**Fig 10B, ii**). In this model, SL1 acts as an attenuator of readthrough structure formation in the absence of CP translation and presumably contributes to the regulation of readthrough levels. Following the red-mediated nucleation of the interaction, additional secondary interactions, such as the orange (**Fig 10C**) or the pink would form in turn and lead to the assembly of an active readthrough structure (**Fig 10D**).

Not depicted in **Fig 10D** is the potential formation of an additional interaction involving the green partner sequences in the PRTE and DRTE. This pairing would extend the helical region at the base and could help to stabilize the structure via coaxial stacking with the orange helix (**Fig 10D**). However, this green interaction would need to be temporary and disengage during ribosome readthrough, so as to allow for the necessary spacer distance (~7–9 nt) between the stop codon and the base of the readthrough structure [1]. Either with or without the involvement of this latter interaction, the active RNA structure, postulated to be that depicted in **Fig 10D**, would then be able to efficiently trigger CP stop codon readthrough, presumably by increasing utilization of near cognate tRNAs or decreasing recruitment of release factors by an unknown mechanism [1]. Active translation of the RTD coding region would cause disruption of PRTE/DRTE interactions and their local RNA structures. Accordingly, for subsequent rounds of readthrough to occur, ribosome-mediated conversion of SL1 to SL2 would again be required to initiate assembly of an active readthrough structure (**Fig 10A and 10B**). It is also noted that the readthrough structure folding process described could also involve other protein factors, such as RNA chaperones and/or RNA helicases.

## Conclusion

This study has provided the first higher-order RNA models for readthrough structures in the Enamovirus and Polerovirus genera. Compelling experimental evidence demonstrating the importance of long-distance RNA-RNA interactions in the formation of these structures was also presented. Compared to other readthrough structures, the proposed structure for PEMV1 is arguably the most elaborate readthrough signal reported to date, and its suggested folding pathway, as well as that for PLRV, provide new insights into readthrough structure assembly. Collectively, these findings significantly advance our understanding of the strategies used by viruses to mediate the production of essential readthrough proteins.

## Materials and methods

### cDNA preparation

Standard PCR-based site-directed mutagenesis was utilized for introducing nucleotide substitutions in different parts of full-length PEMV1 genome (gene bank: NC_003629.1) and PEMV1 sg mRNA. Cloned cDNA of the full-length PEMV1 genome [32,45] (Kindly provided by W. Allen Miller, Iowa State University) was used to create genomic mutants, as well as wt PEMV1 sg mRNA and its mutant derivatives. All viral mutants utilized in this study were sequenced to confirm that only the intended modifications were present.

Full-length PEMV1 genome construct gHA, contained three tandem HA-tag sequences (UACCCAUACGAUGUUCCAGAUUACGCU) introduced at the N-terminal region of CP ORF (genome coordinates 4015–4095, immediately downstream from the first 6 codons of CP). gHA was then utilized as a backbone to insert PRTE-DRTE compensatory nucleotide substitutions, thereby creating gHA7, gHA8 and gHA9.

Mutants constructed to investigate CP-RTD production from the PLRV sg mRNA [51] were derived from PLRV genome cDNA (gene bank: KP090166.1) that was kindly provided by Michelle Heck (Cornell University).

### Synthesis of viral RNAs in vitro

All of the PEMV1 genome and sg mRNA constructs investigated in this study contained a T7 promoter at the 5′-end of the viral sequence and a unique PstI restriction enzyme cut site at its 3′-end. PstI-linearized wt and mutant clones were treated with T4 DNA polymerase (NEB) to remove the 3′-overhang left after PstI cleavage and then were transcribed in vitro using AmpliCap-Max T7 High Yield Message Maker Kit (Cellscript) to create 5′-capped sg RNAs and MessageMax T7 ARCA-Capped Message Transcription Kit (Cellscript) to create 5′-capped genomic RNAs, both with authentic viral 3′ ends.

The PLRV sg mRNA constructs utilized in this study contained a T7 promoter at the 5′-end of the viral sequence and a unique 3′-terminal ScaI restriction enzyme cut site. ScaI-linearized wt and mutant cDNAs were transcribed in vitro using AmpliCap-Max T7 High Yield Message Maker Kit (Cellscript) to create 5′-capped sg mRNAs with authentic viral 3′ ends.

### In vitro translation assays

To test readthrough levels of CP-RTD, 0.5 pmol of 5′-capped transcripts of wt or mutant PEMV1 sg mRNAs (sub-saturating levels) were incubated in wheat germ extract (wge, Promega) in the presence of [^35^S]-Methionine at 25°C for 1 hr according to the manufacturer’s instructions, except that the concentration of KOAc was increased to 133 mM for each reaction to optimize translation and readthrough efficiency. The viral proteins translated during the incubation were detected and quantified through 12% SDS-PAGE and phosphorimaging, respectively [52,53]. Imaging was carried out using Typhoon FLA 9500 Variable Mode Imager (GE Healthcare). QuantityOne software (BioRad) was used to quantify protein bands, from which ratios of the readthrough product CP-RTD and the pre-readthrough product CP were calculated for each tested mRNA. Percentages of the mutant ratios relative to the wt ratio were determined and used as relative readthrough levels (Rel. RT). Three independent repeats were carried out for each of the in vitro translation experiment and means with standard errors (SE) were calculated.

The same steps were followed as above for obtaining readthrough levels of CP-RTD from PLRV sg mRNAs in vitro, except that 0.4 pmol of 5′-capped transcripts (sub-saturating levels) was used per in vitro translation reaction.

### Pea protoplast transfection

Pea protoplasts were isolated from 12-day old, fully expanded *Pisum sativum* leaves by first removing the lower epidermis and then incubating the remaining tissue in a cellulase mixture at 26°C for 4 hours [54,55]. Two million protoplasts were transfected with 20 μg of 5′-capped PEMV1 transcripts using polyethylene glycol (PEG 1450) and CaCl_2_ and incubated at 22°C for 40 hours under constant fluorescent light [55]. After the incubation, one half of the infection was used for total protein isolation and western blotting and the other half for total nucleic acid extraction and Northern blotting.

### Western blotting

Total proteins were separated by 12% SDS-PAGE and transferred to membrane (Amersham Hybond P 0.45 PVDF). Ponceau S staining was carried out for visualizing total proteins and confirming equal loading and transfer prior to proceeding with blotting. HA-tagged CP and CP-RTD were detected by blotting with Anti-HA-peroxidase high affinity (3F10) rat monoclonal antibodies (Roche) at 1:2000 dilution. CP and CP-RTD bands were detected using ECL Select western blotting detection reagent (GE Healthcare) and captured through MicroChemi imager (DNR Bio-Imaging Systems). Detected viral protein bands were quantified using *QuantityOne* software. Three independent repeats of pea protoplast infections/western blotting were carried out and means with SE were calculated. Rel. RT levels were calculated as described for in vitro translation assays.

### Northern blotting

Total nucleic acids were extracted from infected protoplasts, separated by agarose gel electrophoresis, and transferred to nylon membrane (Hybond-N^+^, Amersham), after ensuring even loading via monitoring rRNA levels. Coding sense PEMV1 genome and sg mRNA were detected by blotting with nine oligonucleotide probes 5′-end-labeled with [γ -^32^P] complementary to both the genome and sg mRNA (genome coordinates: 4004–4031, 4324–4359, 4401–4434, 4681–4708, 4749–4780, 4936–4968, 5122–5389, 5406–5439, 5679–5703). Northern blots, from three independent repeats, were captured using Typhoon FLA 9500 Variable Mode Imager and viral RNA bands were quantified using *QuantityOne* software. Sg mRNA levels of each mutant PEMV1 were calculated to generate average values with SE.

### SHAPE RNA structure analysis

Selective 2′-hydroxyl acylation analyzed by primer extension (SHAPE) was performed and the data was used to model the RNA secondary structures of PRTE and DRTE regions in full-length PEMV1 sg mRNA, as described previously [33,6,53]. SHAPE was carried out using 1-methyl-7-nitroisatoic anhydride (1M7) that modifies flexible (*i*.*e*. single stranded) nucleotides. Two primers, fluorescently labeled at their 5′-ends, one complementary to a region downstream from the PRTE (genome coordinates– 4828–4857) and the other to a region downstream from DRTE (genome coordinates –5504–5533), were used for primer extension reactions following 1M7 treatment of wt PEMV1 sg mRNA. After fluorescent capillary electrophoresis of the products of primer extension, the raw data was analyzed using the *ShapeFinder* software [56] to generate relative reactivities for each nucleotide. These reactivity values were normalized against the ten highest reactivities in the pool. The SHAPE experiment was performed twice, with consistent results, and averaged values of the two repeats were used for secondary structure prediction. The *RNAStructure* web server was used [34] to combine SHAPE reactivity data (slope = 1.8 kcal/mol; intercept = -0.6 kcal/mol) with thermodynamic prediction to generate secondary structure models of PEMV1 PRTE and DRTE in the sg mRNA context. *RNA2Drawer* software was utilized to draw RNA secondary structure models depicted throughout the paper [57].
